# The Prevention of Seroma Formation Following Modified Radical Mastectomy by Intravenous Hydrocortisone Injection

**DOI:** 10.7759/cureus.55017

**Published:** 2024-02-27

**Authors:** Sayyam Fatima, Muhammad Salman Shafique, Bushra Shabana, Sumaira Nawaz, Jahangir S Khan, Syed Waqas Hasan

**Affiliations:** 1 Department of Surgical Unit I, Holy Family Hospital, Rawalpindi, PAK; 2 Department of General Surgery, Chesterfield Royal Hospital, Chesterfield, GBR

**Keywords:** modified radical mastectomy, randomized controlled trial, seroma, hydrocortisone, breast carcinoma

## Abstract

Introduction

Seroma formation is the most common complication after modified radical mastectomy (MRM). It leads to increased pain and discomfort, potentially prolonging morbidity and treatment. Various treatment modalities are being used to decrease the incidence of seroma formation. The objective of this study was to compare intravenous hydrocortisone injection versus placebo in patients undergoing MRM in terms of frequency of post-operative seroma formation.

Methods

This randomized, double-blinded, placebo-controlled study was conducted at Surgical Unit-I, Holy Family Hospital, Rawalpindi, Pakistan from January 2021 to December 2021. A total of 152 female patients were randomly assigned to each of the study and placebo groups. Group I patients received 100 mg of hydrocortisone intravenously while group II patients received one ml of 0.9% normal saline intravenously prior to induction of general anesthesia for MRM. The incidence of seroma formation after 10 days of MRM and total drain volume till their removal was measured in all patients.

Results

The mean age was 48.42±10.15 in Group I, while it was 47.67±10.75 in Group II. Mean drain output till removal was 99.14±31.01 ml in the hydrocortisone group and 177.57±63.37 ml in the placebo group. Forty-eight patients developed seroma (31.58%), of whom nine received intravenous hydrocortisone and 39 received normal saline (P=0.000).

Conclusion

Intravenous hydrocortisone is effective in terms of frequency of post-operative seroma formation as compared to placebo in patients undergoing MRM.

## Introduction

Carcinoma of the breast is the most common cancer among females worldwide [1] and accounts for about 20% of deaths among women [2]. Its incidence is increasing in Asian countries like Pakistan, where it is responsible for about 34.6% of total cancer patients in the female population [3]. The management of breast cancer involves a multidisciplinary approach. Surgery includes breast conservation surgery with radiotherapy of remaining breast tissue or mastectomy with axillary lymph node clearance [4]. However, modified radical mastectomy (MRM) is performed with the intent to cure, and less than 1% mortality is attributed to this procedure [3].

The most common and avoidable complication after mastectomy and axillary lymph node dissection is seroma formation, with a frequency ranging from 3% to 85% [5]. It is the collection of serous fluid underneath the skin flaps or in the axillary space, generally beginning on the seventh post-operative day with a peak on the eighth day, ultimately declining over the next few days [6].

Seroma accounts for impaired wound healing, prolonged hospitalization, and substantial morbidity, thereby reducing the quality of life with an ultimate delay in adjuvant therapy [2]. An acute inflammatory exudative response is the basis of surgical trauma which is supposed to play a critical role in the pathogenesis of seroma formation. The presence of proteinases, proteinase inhibitors, cytokines and growth factors in the seroma fluid support this fact [5]. Several techniques have been employed for preventing seroma formation with differing success rates which include shoulder immobilization, prolonged use of suction drains, flap fixation, and perioperative use of tranexamic acid [6].

The use of steroids in decreasing the inflammatory response has been documented in abdominal surgeries, colonic resections, head and neck, plastic, and cardiac surgery with promising results [7]. As a result, steroids like hydrocortisone can prove to be potent anti-inflammatory agents in the prophylaxis of post-MRM seroma formation among breast cancer patients [6]. Steroids are strongly recommended in terms of their cost-effectiveness, ease of use, and safety. In the trial performed by Talha A et al., post-mastectomy seroma occurred in 5% (two out of 40) of patients in the hydrocortisone group compared to 20% (eight out of 40) in the placebo group, with a significant p-value of < 0.005 [2].

This study will determine whether intravenous administration of hydrocortisone at the dose provided is effective in preventing post-MRM seroma formation in breast cancer patients. This will be a simple and effective way to avoid this complication of mastectomy and can easily be incorporated into our management plan. Prevention of seroma formation would lead to a significant reduction in morbidity and will ensure early recovery of the patient.

This article was previously presented as an oral presentation at the 5th Annual Resident Research Conference, Rawalpindi Medical University, Rawalpindi, Pakistan in December 2022, and the abstract was then published in their abstract book.

## Materials and methods

This randomized, double-blinded, placebo-controlled trial was conducted at the Department of Surgical Unit-I, Holy Family Hospital, Rawalpindi, Pakistan from January 2021 to December 2021. It was approved by the Institutional Research Forum and the Research and Ethical Committee of Rawalpindi Medical University, and the trial was then registered under the Registration Number: 137/IREF/RMU/2020. Informed written consent was obtained from all patients, who were kept blinded to the group allocated throughout the study.

**Figure 1:**
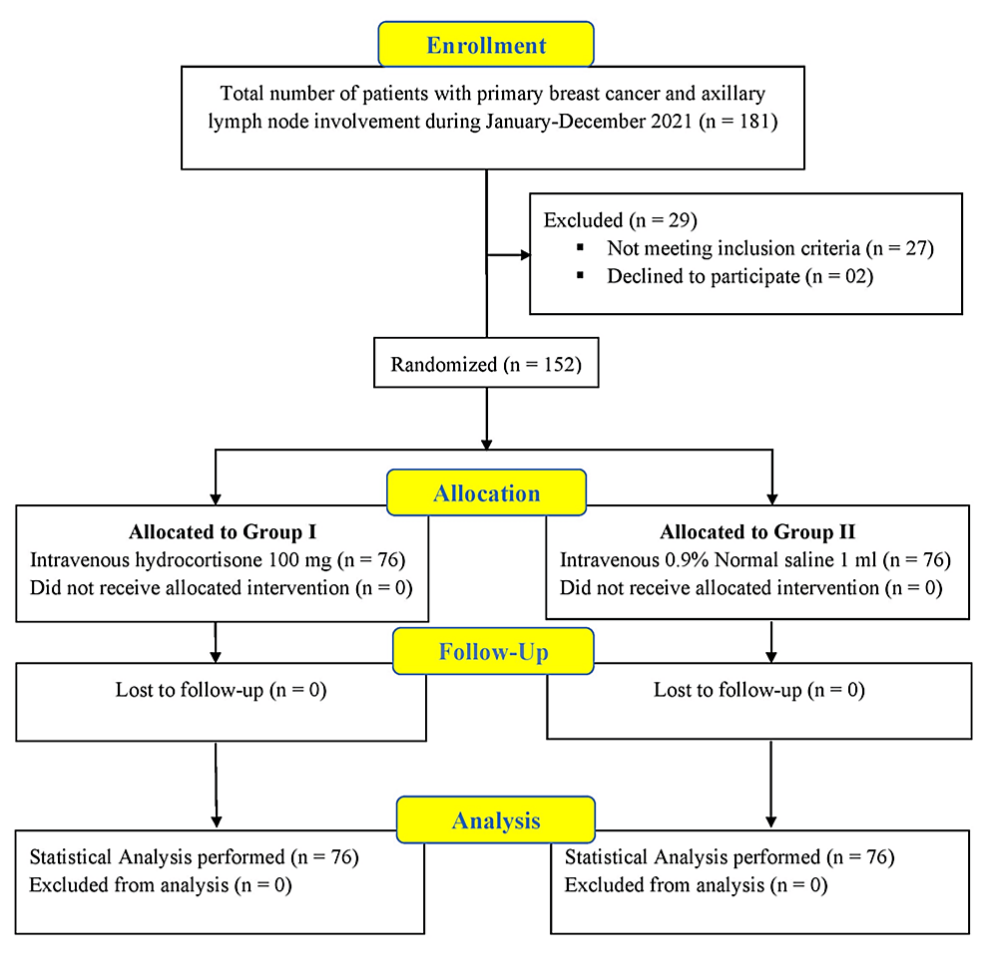
Consort flowchart for patient selection

One hundred and fifty-two (152) patients, who met the inclusion criteria, were enrolled in the study (Figure 1). The sample size was calculated using the WHO calculator. All female patients aged 18 to 70 years, with primary breast cancer and axillary lymph node involvement were included and scheduled for MRM on an elective basis. Patients were excluded from the study if they had stage IV breast cancer, had evidence of local infection, history of previous axillary surgery in the last six months, or had been treated with steroids within the last month before surgery, including inhalational products. All those patients who were pregnant, allergic, or hypersensitive to hydrocortisone were omitted from the study.

The lottery method was employed to randomly assign included patients to one of two groups. The envelope was opened in the operation theatre by the assisting theatre staff prior to induction of general anesthesia for surgery to indicate which patient is to receive 100 mg hydrocortisone by intravenous route (Group I) or one ml of 0.9% normal saline intravenously (Group II). In both groups, the choice of prophylactic antibiotics, surgical technique, and the team of surgeons performing the procedure were standardized irrespective of their randomization. Mastectomy flaps were raised with the use of monopolar diathermy and sharp dissection was performed for axillary clearance. Negative suction was maintained for drains, which were then removed when the daily drainage volume was less than 30 ml. Compression dressing was applied for 10 days, and early arm physiotherapy was started for all patients. Total drain volume till their removal was recorded in the postoperative period. The primary outcome measure was postoperative seroma formation, which was assessed by a doctor on an outdoor basis after 10 days of MRM. All the data was recorded in the specially designed performa. Confounding variables were taken into consideration and bias was removed by randomization.

Data was analyzed using the Statistical Package for the Social Sciences (IBM SPSS Statistics for Windows, IBM Corp., Version 22.0, Armonk, NY). Descriptive statistics were calculated for both qualitative and quantitative variables. Seroma formation was expressed as frequencies and percentages. Mean +/- standard deviation was calculated for age and total fluid drainage. The difference in the frequency of postoperative seroma formation between the two study groups was analyzed using the Chi-square test. Qualitative variables were presented through tables and figures. Stratification was done with respect to age. The Chi-square test was applied after stratification. A p-value of <0.05 was considered statistically significant.

## Results

From January 2021 to December 2021, 152 patients were enrolled on a non-probability consecutive sampling basis. Baseline characteristics among the two study groups are listed in Table 1.

**Table 1:** Characteristics of the patients

Characteristics	Hydrocortisone	Normal saline
	N = 76	N = 76
Age (years)	48.42 ± 10.15	47.67 ± 10.75
Duration of symptoms (months)	15.45 ± 12.27	17.72 ± 16.89
Operative time (minutes)	119.74 ± 25.62	108.03 ± 20.83
Therapeutic intervention for seroma - No. (%)	03 (3.94%)	42 (96.06%)

Seventy-six patients received 100 mg of intravenous hydrocortisone (Group I), and 76 patients received one ml of intravenous 0.9% normal saline (Group II). Our study population was in the age group of 28 to 70 years. The mean age of patients in our study was 48.05 ± 10.43 years. The age distribution of the patients is shown in Table 2.

**Table 2:** Age distribution of the patients

Age (years)	Number	Percentage
21-30	07	4.6
31-40	46	30.3
41-50	42	27.6
51-60	49	32.2
61-70	08	5.3

**Figure 2:**
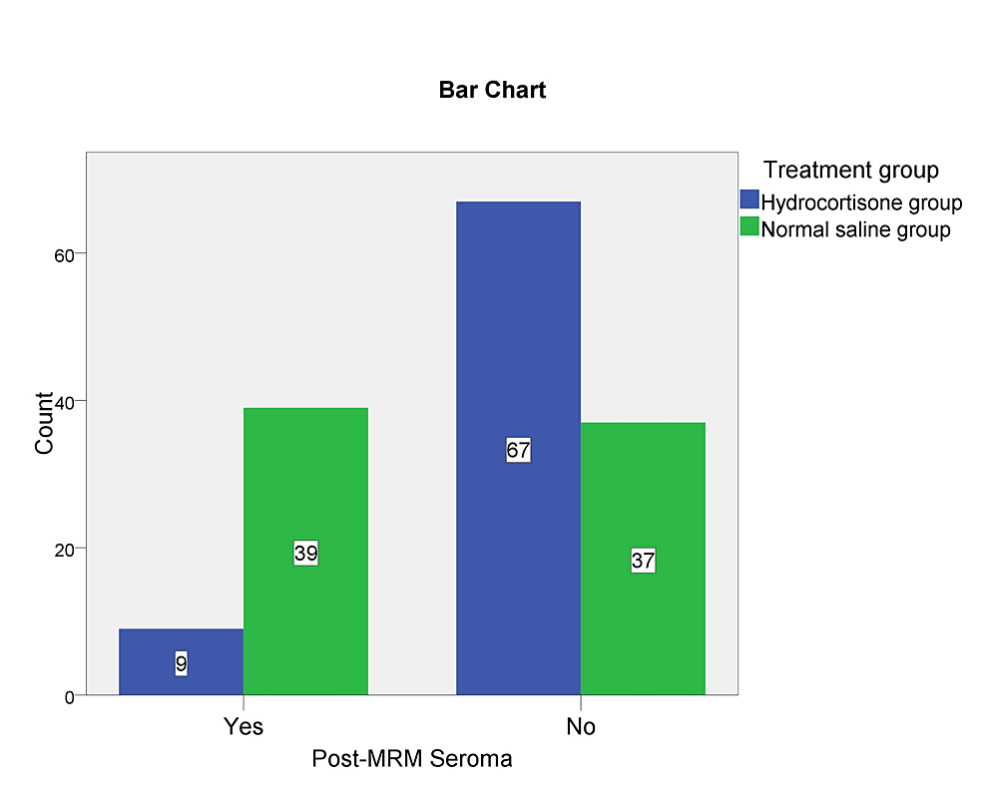
Post-MRM seroma MRM: modified radical mastectomy

After surgery, the mean drain output was 99.14 ± 31.01 ml in the hydrocortisone group and 177.57 ± 63.37 ml in the control group. The difference in mean drain output between the two groups was statistically significant (P = 0.000). After 10 days of MRM, seroma formation occurred in 48 patients (31.58%), nine belonged to the hydrocortisone group and 39 were from the control group (Table 3, Figure 2). Intravenous hydrocortisone was effective in preventing post-MRM seroma formation (P = 0.000).

**Table 3:** Post-MRM seroma and P-value MRM: modified radical mastectomy

Treatment group	Post-MRM seroma							
	N		Mean		Std. Deviation		P-value	
Hydrocortisone		76		1.88		0.196		0.000
Normal Saline		76		1.48		0.437		

Postoperative seroma formation between the two study groups was found to be statistically significant in age groups 31-40, 41-50 and 51-60 years, when stratified with respect to age. This is shown in Table 4.

**Table 4:** Comparison of postoperative seroma formation between the study groups with respect to age MRM: modified radical mastectomy

Age (years)	Treatment group	Post-MRM seroma		Total	P-value
		Yes	No		
21-30	Hydrocortisone	00	02	02	0.495
	Normal Saline	01	04	05	
	Total	01	06	07	
31-40	Hydrocortisone	02	18	20	0.001
	Normal Saline	15	11	26	
	Total	17	29	46	
41-50	Hydrocortisone	03	23	26	0.006
	Normal Saline	08	08	16	
	Total	11	31	42	
51-60	Hydrocortisone	03	23	26	0.000
	Normal Saline	14	09	23	
	Total	17	32	49	
61-70	Hydrocortisone	01	01	02	0.346
	Normal Saline	01	05	06	
	Total	02	06	08	

## Discussion

Seroma formation is the most common and grave complication of MRM [8]. The underlying mechanism of post-MRM seroma formation is thought to be due to the disruption of lymphatic channels and the creation of a dead space during flap elevation. Several factors are found in high concentration in seroma fluid such as IgG, granulocytes, proteinases, leukocytes, and many other cytokines [9]. There is documented evidence that corticosteroids, such as hydrocortisone, possess strong anti-inflammatory properties, thereby preventing the accumulation of fluid at the site of the surgical area [2].

The aim of this study was to evaluate the effect of intravenously administered hydrocortisone as a prophylactic agent for post-MRM seroma formation. The results, discussed in detail, are consistent with the internationally published literature.

Our study showed that 100 mg of hydrocortisone injection, administered at the time of induction for MRM, can reduce the incidence of a common but troublesome complication i.e., post-MRM seroma [2,3,8].

Corticosteroid-associated reduction of post-MRM seroma incidence was not observed in the study by Okholm and Axelsson [10]. The reason for a negative correlation between corticosteroid administration and lower incidence of seroma formation may be linked to the fact that the inflammatory response after MRM is even more marked than after head and neck surgery. A lower dose of corticosteroid was used in the study, which could be related to the failure of a pronounced effect, leading to more seroma formation.

A study conducted by Schulze et al. [11] showed the positive anti-inflammatory effect of a single preoperative high-dose steroid in patients undergoing open colonic resection. Similar success has been described by Taghizadeh et al. [12] in seroma management. A single dose of glucocorticoid was effective in significantly reducing the total number of aspirations and total volume aspirated.

The overall incidence of post-MRM seroma formation in the present study was 31.58%, with a normal saline (control) group incidence of 51% and a hydrocortisone (drug) group incidence of 12% with a significant P-value (P=0.000). Comparable results were obtained from the trials conducted by Talha et al. [2], Khan [3], and Qvamme et al. [5]. Another Indian study concluded that the injection of hydrocortisone significantly reduced seroma formation [13]. The incidence of post-MRM seroma in the placebo group of the present trial was much greater than that of previous studies in which the mean rate of seroma formation was 20% and 18.75%.

Following MRM, the mean drain output was noted to be 99.14 ± 31.01 ml in the hydrocortisone group, whereas the mean drainage volume in the placebo group was 177.57 ± 63.37 ml, with a significant p-value of 0.000. Our results are comparable with the findings of Talha et al. [2], Qvamme et al. [5], and Albatanony et al. [8].

Intravenous administration of corticosteroids significantly reduced the incidence of post-MRM seroma formation, with minimal side effects. It is a safe, feasible, cost-effective, and rewarding treatment modality, especially in hospitals with limited resources.

The main limitation of this study was the small sample size, and consequently, the power of the study was low. Many factors are responsible for seroma causation after MRM, with multiple preventive and therapeutic options available to reduce the morbidity associated with this complication. The authors recommend that this study should be conducted on a large scale, so that the validity of corticosteroids in reducing the incidence of post-MRM seroma formation can be established. This would then be incorporated into the standard management plan, thereby reducing a common and morbid complication of MRM.

## Conclusions

Seroma formation is the most common complication in patients undergoing MRM. It is a pro-inflammatory process that can lead to other serious complications like flap necrosis, skin infection, and delayed wound healing. Intravenous hydrocortisone prior to induction of anesthesia appears to be a useful, cost-effective, and promising technique in terms of frequency of postoperative seroma formation in patients undergoing MRM.

## References

[1] Sharma A, Sharma C, Tickoo S (2017). The effect of radiotherapy following modified radical mastectomy on hand grip strength and pinch grip strength in breast cancer females. SRHU Med J.

[2] Talha A, Ramadan R, Abdelhamid S (2015). Postmastectomy seroma: how much is it affected by serum levels of IL-6 and CRP and how much is it reduced by intravenous hydrocortisone injection?. Egypt J Surg.

[3] Khan MA (2017). Effect of preoperative intravenous steroids on seroma formation after modified radical mastectomy. J Ayub Med Coll Abbottabad.

[4] Runowicz CD, Leach CR, Henry NL (2016). American Cancer Society/American Society of Clinical Oncology Breast Cancer survivorship care guideline. CA Cancer J Clin.

[5] Qvamme G, Axelsson CK, Lanng C (2015). Randomized clinical trial of prevention of seroma formation after mastectomy by local methylprednisolone injection. Br J Surg.

[6] Vijayalakshmi S (2018). A comprehensive study on the effect of injection methylprednisolone in post mastectomy seroma. IAIM.

[7] Axelsson CK, Qvamme GM, Okholm M (2019). Bacterial colonization of seromas after breast cancer surgery with and without local steroid prophylaxis. World J Surg Oncol.

[8] Albatanony AA, Assar AMA, El Balshy MAE (2021). Correlation between C-reactive protein, intravenous hydrocortisone, systemic tranexamic acid, and post mastectomy seroma. Int Surg J.

[9] Prajapati S, Ramasamy S, Vats M, Neogi S, Kantamaneni K, Tudu SK (2021). Effect of octreotide on lymphorrhea in patients after modified radical mastectomy for carcinoma breast: a randomized controlled trial. Cureus.

[10] Okholm M, Axelsson CK (2011). No effect of steroids on seroma formation after mastectomy. Dan Med Bull.

[11] Schulze S, Andersen J, Overgaard H (1997). Effect of prednisolone on the systemic response and wound healing after colonic surgery. Arch Surg.

[12] Taghizadeh R, Shoaib T, Hart AM, Weiler-Mithoff EM (2008). Triamcinolone reduces seroma re-accumulation in the extended latissimus dorsi donor site. J Plast Reconstr Aesthet Surg.

[13] Shyamsundar R (2020). A study on local injection of methylprednisolone acetate to prevent seroma formation after mastectomy (thesis). Madras (Ind): Madras Medical College.

